# In vitro and in vivo potentialities for cartilage repair from human advanced knee osteoarthritis synovial fluid-derived mesenchymal stem cells

**DOI:** 10.1186/s13287-018-1071-2

**Published:** 2018-11-28

**Authors:** Paul Neybecker, Christel Henrionnet, Elise Pape, Didier Mainard, Laurent Galois, Damien Loeuille, Pierre Gillet, Astrid Pinzano

**Affiliations:** 1 UMR 7365 CNRS-UL, IMoPA (Ingénierie Moléculaire et Physiopathologie Articulaire), Biopôle de l’Université de Lorraine, Campus Brabois-Santé, 9 Avenue de la Forêt de Haye, BP 20199, 54505 Vandœuvre-Lès-Nancy, France; 2 Service de Chirurgie Orthopédique, Traumatologique et Arthroscopique, CHRU Nancy, 29 Avenue du Maréchal de Lattre de Tassigny CO 60034, F54035 Nancy, France; 3 Service de Rhumatologie, CHRU de Nancy, Hôpitaux de Brabois, Bâtiment des Spécialités Médicales, 5 rue du Morvan, F54511 Vandœuvre-lès-Nancy, France

**Keywords:** Synovial fluid, Stem cells, Collagen sponge, Cartilage, Tissue engineering

## Abstract

**Background:**

Mesenchymal stem cells (MSCs) are found in synovial fluid (SF) and can easily be harvested during arthrocentesis or arthroscopy. However, SF-MSC characterization and chondrogenicity in collagen sponges have been poorly documented as well as their hypothetical in vivo chondroprotective properties with intra-articular injections during experimental osteoarthritis (OA).

**Methods:**

SF-MSCs were isolated from human SF aspirates in patients suffering from advanced OA undergoing total knee joint replacements. SF-MSCs at passage 2 (P2) were characterized by flow cytometry for epitope profiling. SF-MSCs at P2 were subsequently cultured in vitro to assess their multilineage potentials. To assess their chondrogenicity, SF-MSCs at P4 were seeded in collagen sponges for 4 weeks under various oxygen tensions and growth factors combinations to estimate their gene profile and matrix production. Also, SF-MSCs were injected into the joints in a nude rat anterior cruciate ligament transection (ACLT) to macroscopically and histologically assess their possible chondroprotective properties,.

**Results:**

We characterized the stemness (CD73+, CD90+, CD105+, CD34−, CD45−) and demonstrated the multilineage potency of SF-MSCs in vitro. Furthermore, the chondrogenic induction (TGF-ß1 ± BMP-2) of these SF-MSCs in collagen sponges demonstrated a good capacity of chondrogenic gene induction and extracellular matrix synthesis. Surprisingly, hypoxia did not enhance matrix synthesis, although it boosted chondrogenic gene expression (*ACAN*, *SOX9*, *COL2A1*). Besides, intra-articular injections of xenogenic SF-MSCs did exert neither chondroprotection nor inflammation in ACLT-induced OA in the rat knee.

**Conclusions:**

Advanced OA SF-MSCs seem better candidates for cell-based constructs conceived for cartilage defects rather than intra-articular injections for diffuse OA.

## Background

Articular cartilage has a very limited self-healing potential. Articular cartilage defects are a common problem in orthopedic surgery and can lead to osteoarthritis (OA). Many cells-based treatment strategies for cartilage lesions were developed, and autologous chondrocyte implantation (ACI) is a standard treatment for focal chondral lesions repair [1]. However, ACI has disadvantages and leads to the formation of fibrotic and hypertrophic cartilage and possible donor site morbidity by risk of OA development [2].

For cartilage engineering, chondrocytes, the unique specialized resident cells of cartilage, seem to be the best candidate. However, significant inconvenients of this contingent can be listed: cartilage biopsy is an invasive act, the number of chondrocytes is very low, and there is a rapid dedifferentiation during the monolayer cell expansion [3, 4]. For two decades, mesenchymal stem cells (MSCs) are studied as an interesting alternative source of cells for cartilage engineering due to their self-renewal capacity, their accessibility, and their multilineage differentiation capacity. MSCs can be isolated from different adult tissues such as bone marrow, adipose tissue, and synovium or from fetal tissues such as Wharton’s jelly [3].

The synovial fluid (SF) also represents a novel source of cells for cartilage therapeutic approaches. In fact, SF is easily harvested during arthrocentesis, arthroscopy, or knee surgery. Many studies show the multilineage differentiation of cells from SF in human [5, 6], in horse [7], or in bovine SF [8]. Moreover, it was recently demonstrated that the chondrogenic differentiation is equivalent between equine bone marrow MSCs and equine SF-MSCs and even more potent in pigs [9, 10]. All these studies confirmed the stemness of SF cells, so-called SF-MSCs, in accordance with the criteria of the International Society for Cellular Therapy [11].

However, the SF-MSCs lineage of is not yet clearly elucidated but it is commonly accepted that their origin comes from different tissues in the knee, like cartilage or notably synovium tissue [12]. SF-MSCs are found at higher concentration in OA joint and in joint with meniscal or ligaments injuries than in healthy joints [6, 8, 12–14]. These SF-MSCs display several advantages: (i) they can be easily collected by knee joint puncture, a minimally invasive act, during the SF effusion; (ii) they allow to use autologous cells; (iii) they have already an articular commitment inherent to their joint origin and integrated joint constraints (hypoxia, mechanical stresses); and (iv) they show immunoregulatory and immunosuppressive properties [13, 15].

Concerning cartilage engineering, apart the cell source and the scaffold used, the quality of a chondral implant relies especially on two conditions: (i) the cocktail of growth factors used and (ii) the oxygen tension. Different studies showed that TGF-β family stimulates MSCs-driven chondrogenic differentiation [16–18]. On the other hand, many papers have demonstrated that a low-oxygen tension (3–5%) promotes MSC-driven chondrogenic differentiation [19–21].

The objective of this study is to explore the potentialities of an original cell source of MSCs derived from synovial fluid (SF-MSCs) from OA patients undergoing total knee joint replacement (i) to produce cartilage tissue-engineered substitutes to treat focal lesions of cartilage and (ii) the potentialities of intra-articular (i.a.) injections of SF-MSCs to treat experimental knee OA diffuse lesions in the rat. To this end, we isolated and characterized resident cells present in synovial fluid issued from OA patients. We investigated human OA SF-MSCs differentiation potential to produce cartilaginous tissue-engineered substitutes after 28 days inside 3D collagen sponges under the influence of growth factors (TGF-ß1 and/or BMP-2) and oxygen tension (normoxia or hypoxia). Additionally, we studied the influence of repeated i.a. SF-MSCs injections in a model of anterior cruciate ligament transection in nude rats.

## Methods

### Isolation and expansion of mesenchymal stem cells issued from synovial fluid

Human synovial fluid from the knee was obtained from severe knee OA patients undergoing total knee replacement surgery (Kellgren Lawrence score 3–4). All subjects enrolled in this research have responded to an informed consent which has been approved by our Institutional Committee on Human Research (2014, July 10, #DC-2014-2148), and this protocol has been found acceptable by them.

SF samples were diluted at 1:6 in a proliferation medium and plated in 55-cm^2^ Petri dishes. After 3 or 4 days, the medium was changed to remove the non-adherent cells. The cells were cultured in a proliferation medium containing Dulbecco’s modified Eagle’s medium with low glucose (DMEM-LG, Gibco) supplemented with 10% fetal bovine serum (FBS, Sigma), 1 ng/ml basic fibroblast growth factor (bFGF, Miltenyi Biotec), 1% glutamine (Gibco), and 1% penicillin-streptomycin (Gibco). The dishes were cultured at 37 °C with 5% of humidified CO_2_. The medium was unchanged for the initial 3 days and then changed twice per week until confluence. The non-adherent cells were discarded with sequential changes of medium. When the adherent cells reached around 80% of confluence, MSCs were trypsinized (trypsin-EDTA 0.05% Gibco) and plated at a density of 0.5 × 10^6^ cells/flask. The medium was changed the following day and then every 2–3 days.

During the last passage (P3) before seeding the cells in collagen sponges, a pre-differentiation step was performed. SF-MSCs were cultured with differentiation medium composed of a Dulbecco’s modified Eagle’s medium with high glucose (DMEM-HG, Gibco) supplemented with sodium pyruvate (110 μg/ml), bFGF (1 ng/ml), 1% penicillin-streptomycin (Gibco), and chondrogenic supplements: proline (40 μg/ml, Sigma), l-ascorbic acid-2-phosphate (50 μg/ml, Sigma), and dexamethasone (10^−7^ M, Sigma).

### Flow cytometric analysis of SF-MSCs

At the end of the second passage, the cell samples were washed with a blocking solution (0.5% BSA (A-9667 Sigma Aldrich, France) in 1× PBS) and distributed at 500,000 cells by tube. The cells were centrifuged (300*g*, 5 min) and the pellets were resuspended in an immunoblotting solution containing either different pairs of antibodies [anti CD45 (BD Pharmingen)/anti CD34 (BD Pharmingen), anti CD73 (BD Pharmingen)/anti HLA-DR (Beckman Coulter), anti CD90 (Beckman Coulter)/anti-CD105 (Beckman Coulter)] or 100 μL of blocking solution as our negative control. After 45 min incubation (4 °C, in the dark), the cells were washed with PBS (Gibco) and centrifugated (300*g*, 5 min). Pellets were resuspended in 300 μL of PBS and tubes analyzed to flow cytometer (Gallios, Beckman Coulter).

### Trilineage differentiation potential of SF-MSCs (pellets)

#### Chondrogenesis

After two monolayer passages, SF-MSCs were trypsinized and centrifuged (0.5 × 10^6^ cells per pellet) at 300 g for 10 min in 15 mL tubes. Pellets were cultivated in a chondrogenic medium containing TGF-β1 (10 ng/mL, Miltenyi Biotec) in DMEM-HG (4.5 g/L, Invitrogen) supplemented with l-ascorbic acid 2-phosphate (50 μg/ml, Sigma), sodium pyruvate (100 mM, Invitrogen), proline (40 μg/ml, Sigma), dexamethasone (10^−7^ M, Sigma), 1% penicillin-streptomycin (Gibco), and ITS+premix (Beckton Dickinson) and supplemented with TGF-β1 (10 ng/mL, Miltenyi Biotec). After 28 days of culture, the pellets were fixed in 4% paraformaldehyde (PFA, Sigma) during 24 h at 4 °C, dehydrated, embedded in paraffin, and cut into 5-μm-thick sections with a microtome. Slides were stained with alcian blue to observe proteoglycan content.

#### Adipogenesis

After two monolayer passages, SF-MSCs were seeded in 24-well plates at 5000 cells per well and cultured in the adipogenic medium for 21 days. Adipogenic medium is composed by DMEM-HG (4.5 g/L; Gibco) supplemented with 10% of fetal bovine serum (FBS, Sigma), 2 mM Glutamax (Gibco), 1% penicillin-streptomycin (Gibco) 110 μg/mL sodium pyruvate (Gibco), 1 μM dexamethasone (Sigma), 5 μg/mL insulin (Sigma), 500 μM isobutyl-methyl-xanthine (Sigma), and 0.2 mM indomethacin (Sigma). After 21 days of culture, the cells were washed with PBS twice, fixed with 4% paraformaldehyde during 30 min, and rinsed with water with 60% isopropanol and were stained with Oil red O (Sigma) during 30 min to detect lipid vacuoles.

#### Osteogenesis

After two monolayer passages, SF-MSCs were seeded in 24-well plates at 10,000 cells per well and cultured in osteogenic medium for 21 days. Osteogenic medium is composed of high-d-glucose (4.5 g/L) DMEM (Invitrogen) supplemented with 10% of fetal bovine serum (FBS, Sigma), 2 mM glutamine (Gibco), 110 μg/mL sodium pyruvate (Gibco), 1% penicillin-streptomycin (Gibco), 50 μg/mL l-ascorbic acid 2-phosphate (Sigma), 100 nM dexamethasone (Sigma), and 10 mM β-glycerophosphate (Sigma). After 21 days of culture, the cells were washed with PBS (Gibco) and fixed with 4% paraformaldehyde during 30 min and calcium deposits were stained with Alizarin Red S (Sigma) during 5 min.

### 3D cell seeding in collagen sponges

The biomaterials used are made of type III and I collagen sponges (95% of type I collagen; diameter 5 mm, thickness 2 mm) manufactured by “Symatèse Biomatériaux” company (Chaponost, France). The cells were seeded into sponges at P4 (passage 4) at the density of 0.5 million cells per sponges and plated in a 48-well plate at 37 °C in humidified atmosphere containing 5% CO_2_ (*v*/*v*). The sponges were divided into two groups and cultured in normoxia (20% O_2_, *v*/*v*) or in hypoxia (5% O_2_, *v*/*v*). The chondrogenic differentiation medium was DMEM-HG (4.5 g/L) supplemented with 1% glutamine (Gibco), 1% streptomycin and penicillin (Gibco), 40 μg/ml proline (Sigma), 50 μg/ml l-ascorbic acid-2-phosphate (Sigma), 10^−7^ M dexamethasone (Sigma), and 1% sodium pyruvate (Gibco). For both groups (normoxia and hypoxia), the sponges were treated from day 3 either (i) with ITS 1% (Insulin-Transferrin-Selenium; ITS+premix, BD) as control, versus (ii) ITS 1% + 100 ng/mL BMP-2 (Miltenyi Biotec) or (iii) ITS 1% + 10 ng/mL TGF-β1 (Miltenyi Biotec) or (iv) ITS + TGF-ß1 + BMP-2 in combination. The sponges were harvested on D28 for analysis.

### Real-time RT-PCR analysis

After 28 days for each condition, the chondrogenic differentiation of SF-MSC was assessed by mRNA expression levels of RPS29 (housekeeping gene—NM_001032), type II collagen (*COL2A1*—NM_001844), isoform 2B (*COLIIB*—NM_033150.2), type I collagen (*COL1A1*—NM_000088.3), aggrecan (*ACAN*—NM_001135), versican (*VCAN*—NM_001164098), *COMP* (NM_000095), SRY (sex determining region Y)-box 9 (*SOX9*—NM_000346), runt-related transcription factor 2 (*RUNX2*—NM_001278478) and osteocalcin (*BGLAP*—NM_199173). The total cell RNA was extracted using the RNeasy mini kit (QIAGEN), according to the manufacturer’s instructions. Quantitative real-time polymerase chain reaction primers are detailed in Table 1. The RNA was quantified spectrophotometrically and reverse transcripted with the iScriptTM cDNA Synthesis Kit (Bio-Rad) according to the manufacturer’s instructions. After cDNA synthesis, quantitative RT-PCR was performed with a Lightcycler (Applied Biosystems). cDNA of each gene was obtained according to the manufacturer’s instructions (Qiagen, purification kit), quantified, then diluted to obtain a standard curve of the 10^−3^ to 10^−6^ μg/mL range, which allows the quantification of the expression of the gene of interest. For each condition, the signal of the *RPS29* housekeeping gene was determined once for each cDNA sample, and this was used to normalize the results for all other genes. For standardization of gene expression levels, results were expressed as a ratio of the mRNA level of each gene of interest over the *RPS29* gene.

**Table 1:** Primers used for RT-qPCR

Gene		Primer sequence	Annealing temperature (°C)	Amplicon size (bp)	Accession number
RPS29	Fwd	5′-AGATGGGTCACCAGCAGCTGTACTG-3′	60	73	NM_001032
	Rev	5′-AGACACGACAAGAGCGAGAA-3′			
COL2A1	Fwd	5′-ATGACAATCTGGCTCCCAAC-3′	55	200	NM_001844
	Rev	5′-GAACCTGCTATTGCCCTCTG-3′			
COL2B	Fwd	5′-GCATGAGGGCGCGGTAGAGA-3′	70	195	NM_033150.2
	Rev	5′-TGGTCCTGGTTGCCGGACAT-3′			
COL1A1	Fwd	5′-AGGTGCTGATGGCTCTCCT-3′	60	104	NM_000088.3
	Rev	5′-GGACCACTTTCACCCTTGT-3′			
ACAN (Aggrecan)	Fwd	5′-TCGAGGACAGCGAGGCC-3′	63	85	NM_001135
	Rev	5′-TCGAGGGTGTAGCGTGTAGAGA-3′			
VCAN (Versican)	Fwd	5′-TGTTCCTCCCACTACCCTTG-3′	62	122	NM_001164098
	Rev	5′-CTTCCACAGTGGGTGGTCTT-3′			
COMP	Fwd	5′-ACAATGACGGAGTCCCTGAC-3′	60	115	NM_000095
	Rev	5′-TCTGCATCAAAGTCGTCCTG-3′			
SOX9	Fwd	5′-GAGCAGACGCACATCTC-3′	55	118	NM_000346
	Rev	5′-CCTGGGGATTGCCCCGA-3′			
RUNX2	Fwd	5′-CCCGTGGCCTTCAAGGT-3′	60	73	NM_001278478
	Rev	5′-CGTTACCCGCCATGACAGTA-3′			
BGLAP (OSTEOCALCIN)	Fwd	5′-GTGCAGAGTCCAGCAAAGGT-3′			
	Rev	5′-TCAGCCAACTCGTCACAGTC-3′	62	175	NM_199173

### Biochemical analysis of cartilaginous tissue-engineered substitutes for GAG content

Sponge lysates obtained after digestion for molecular biology were used to determine GAGs content. This technique is based on a colorimetric assay using dimethylmethylene blue (DMB, Sigma) dye according to Goldberg’s method [22]. The absorbance was measured at 525 nm with spectrophotometer (Dynatech) and compared to standard curve of chondroitin sulfate from shark cartilage.

### Histological analysis of cartilaginous tissue-engineered substitutes

At D28, the collagen sponges were fixed with 4% PFA for 24 h at 4 °C, embedded in paraffin, and cut into 5-μm thick sections with Leica microtome (RM 2135, Leica). The slides were stained with Hematoxylin-?>Erythrosin- (Sigma) for morphology, with alcian blue (Sigma) for proteoglycan content, with Sirius red (Sigma) for collagen content and observed with polarized light microscopy for collagen networks and visualized on optical microscope (DMD 108, Leica).

### Immunohistochemical analysis of cartilaginous tissue-engineered substitutes

The immunohistochemistry was performed according to LSAB + kit (HRP, Dako) based on avidin-biotin techniques with antibodies for type I and type II collagens. A primary monoclonal antibodies collagen II (6B3, Labvision) was used at the dilution of 1/100. The paraffin-embedded tissue sections of 5 μm were deparaffinized through series of alcohols and treated with pepsin (0.4% *w*/*v* in 0.01 M HCl, pH 2.0) during 30 min at room temperature. Slides were then incubated with hydrogen peroxide block solution for 5 min to block endogenous peroxidase. After washing, 2% BSA solution was applied for 10 min at room temperature to block the unspecific epitopes. The primary antibody was added to each slide, and slides were incubated at room temperature in a humidified chamber for 1 h. Subsequently, the samples were incubated with a biotinylated linked secondary antibody for 45 min at room temperature. The peroxidase-labeled steptavidin was applied at room temperature for 30 min. Substrate-chromogen solution was prepared with diaminobenzidine (DAB, LSAB®+ kit, Dako), incubated to the specimen and monitored under a microscope for the desired stain intensity. Control groups for immunohistochemical analysis were performed under identical conditions on cartilage (see thereafter OA model) and on collagen sponge for positive control or without primary antibodies for negative control. Finally, the sections were counterstained with hematoxylin at 1/5 for 1 min (RAL, France).

### Densitometry of glycosaminoglycans and type II collagen using ImageJ

The slides were observed by light microscopy (DMD 108, Leica). The transmitted light images were recorded and treated by a semi-quantitative purpose made method using image analysis software ImageJ to calculate the staining percentage area. Histological assessment of alcian blue stain and IHC marker for collagen type II was performed. For alcian blue stain, the TIFF images were opened with ImageJ. Then, the image was adjusted to color threshold from hue, saturation, and brightness (HSB) to blue (RGB mode) color space. All the good positions were first detected, and regions of interest (ROI) around each culture conditions were defined. alcian blue-positive regions in each culture were selected automatically by using local threshold. This threshold was identical across all biological and technical replicates within each experiment. For IHC percentage determination, the same procedure aforementioned was utilized with red color segmentation by threshold (RGB mode) image and was adjusted to color threshold set in hue, saturation, and brightness (HSB).

### Rat experimental OA

To avoid any immunological conflict against the xenograft of human SF-MSC, 5-week-old nude male rats (RNU for Rowett nude, Charles River Laboratories) were used in this work. This athymic nude rat is deficient for T cells and shows depleted cell populations in thymus-dependent areas of peripheral lymphoid organs. RNU rats were housed in plastic cages with sawdust bedding that was enriched with nesting material and maintained at 21 °C with 12-h/12-h light/dark cycle. The animals were housed in groups of four animals per individual cage. Discomfort and welfare were evaluated daily by the animal caretakers. Rats were fed a standard diet and had access to tap water ad libitum. European ethical guidelines for the care and use of laboratory animals have been respected throughout the study period. The experimental protocol was accepted by our local animal experimentation committee (CELMEA) the 10th of October 2016, under the reference APAFIS#6624-2016042215241254. RNU rats underwent an anterior cruciate ligament transection (ACLT) under general anesthesia as previously described [23] on D0 (right knee). Simultaneously, a sham group underwent arthrotomy without ACLT. One week (D7) and 2 weeks (D14) after OA surgical induction, a SF-MSC suspension in saline (NaCl) was injected i.a. through the patellar ligament into the right knee joint at a concentration of one million cells/50 μL (ACLT + SF-MSCs, 16 rats). The sham (16 rats) and ACLT (16 rats) were injected with 50 μl of sterile saline only on D7 and D14. Body weight was recorded twice weekly during the experiment. Animals were euthanized by ketamine overdose on D28 (8 sham, 8 ACLT saline, and 8 ACLT SF-MSCs rats) and D56 (8 sham, 8 ACLT saline, and 8 ACLT SF-MSCs rats) under general anesthesia and then the joints collected for histological analysis.

#### Macroscopical analysis

All femurs were observed on a binocular magnifying glass in order to score cartilage lesions. Lesions have been graded as it follows: 0 = normal appearance; 1 = slight yellowish discoloration of the chondral surface; 2 = little cartilage erosions in load-bearing areas; 3 = large erosions extending down to the subchondral bone; and 4 = large erosions with large areas of subchondral bone exposure. Each of the chondral compartments of the knee (the medial and lateral femoral condyles, the medial and lateral tibial plateaus, the patella and the femoral groove) were graded separately and blindly by two observers (AP and CH).

#### Histological scoring

Specimens of femur, tibia, patella, and synovial membrane were fixed separately in formalin (VWR chemical). Femur, tibia, and patella were decalcified in 5% formic acid (Sigma) during 10 days, and all samples were dehydrated by a descending series of ethanol with the use of an automated tissue processing apparatus, embedded in paraffin, and cut into 5-μm-thick sections with Leica microtome (RM 2135, Leica).

*The scoring of OA lesions* was graded on a scale adapted from Mankin’s score by two independent observers (AP and CH) in four compartments: medial and lateral part of the tibia and medial and lateral part of the femur. HES and toluidine blue stainings were used. This score ranged from 0 to 23 per compartment according to:

- The structure (0 = normal, 1 = pannus, 2 = surface irregularities, 3 = clefts to transitional zone, 4 = clefts to radial zone, 5 = clefts to calcified zone, 6 = complete disorganization),
- The cellularity (0 = normal, 1 = diffuse hypercellularity, 2 = cloning, 3 = hypocellularity), safranin O staining (0 = normal, 1 = slight reduction, 2 = moderate reduction, 3 = severe reduction, 4 = no staining),
- The thickness of hypertrophic chondrocyte layer (0 = normal, 1 = moderate decrease, 2 = total decrease), Sirius red staining (0 = normal, 1 = intensification of the superficial layer, 2 = intensification until the middle zone, 3 = intensification until calcified zone),
- The collagen network organization (polarized light microscopy of Sirius red) (0 = normal, 1 = intensification of superficial layer, 2 = intensification until middle zone, 3 = intensification until calcified zone),
- The bone remodeling (0 = none, 1 = present)
- The bone osteolysis (0 = none, 1 = present).

*The inflammation of the synovial membrane* was graded on a scale adapted from Rooney’s score by two independent observers (AP and CH) in four fields of view. Six parameters were studied: (1) number of synovial lining cells, (2) surface fibrin deposition, (3) fibrosis, (4) blood vessel proliferation, (5) perivascular infiltrates, and (6) diffuse infiltrates of lymphocytes.

### Statistical analysis

#### In vitro data

The results were performed for each condition (flow cytometry, multipotency, gene expression, 3D culture) in triplicate for three patients. For standardization of gene expression levels, results were expressed as a ratio of the mRNA level of each gene of interest over the *RPS29* gene at D28 with various oxygen concentrations and presented as mean ± standard error to the mean. The significance was determined in a first step by a one-way ANOVA comparison with a Dunnett’s post hoc test comparing TGFß-1, BMP-2, TGF-ß1 + BMP-2 with our reference (ITS) separately in normoxia and hypoxia. In a second step, a significant interaction was assessed between both normoxia and hypoxia for all condition (ITS, TGFß-1, BMP-2, TGF-ß1 + BMP-2) with a two way ANOVA followed with a Bonferroni’s test to assess the influence of hypoxia versus normoxia for each condition. Statistics analysis were performed with GraphPad Prism®.

#### In vivo data

Data are expressed as mean ± standard error to the mean. For each time point (D28 and D56), eight rats were studied for each condition: sham, ACLT saline, ACLT SF-MSCs. For the three scoring (macroscopy, Mankin’s, and Rooney’s scores), a one-way ANOVA followed by a Bonferroni’s post hoc test was performed. A *p* value less than 0.05 was considered significant.

## Results

### Characterization of human SF-MSCs

**Fig. 1:**
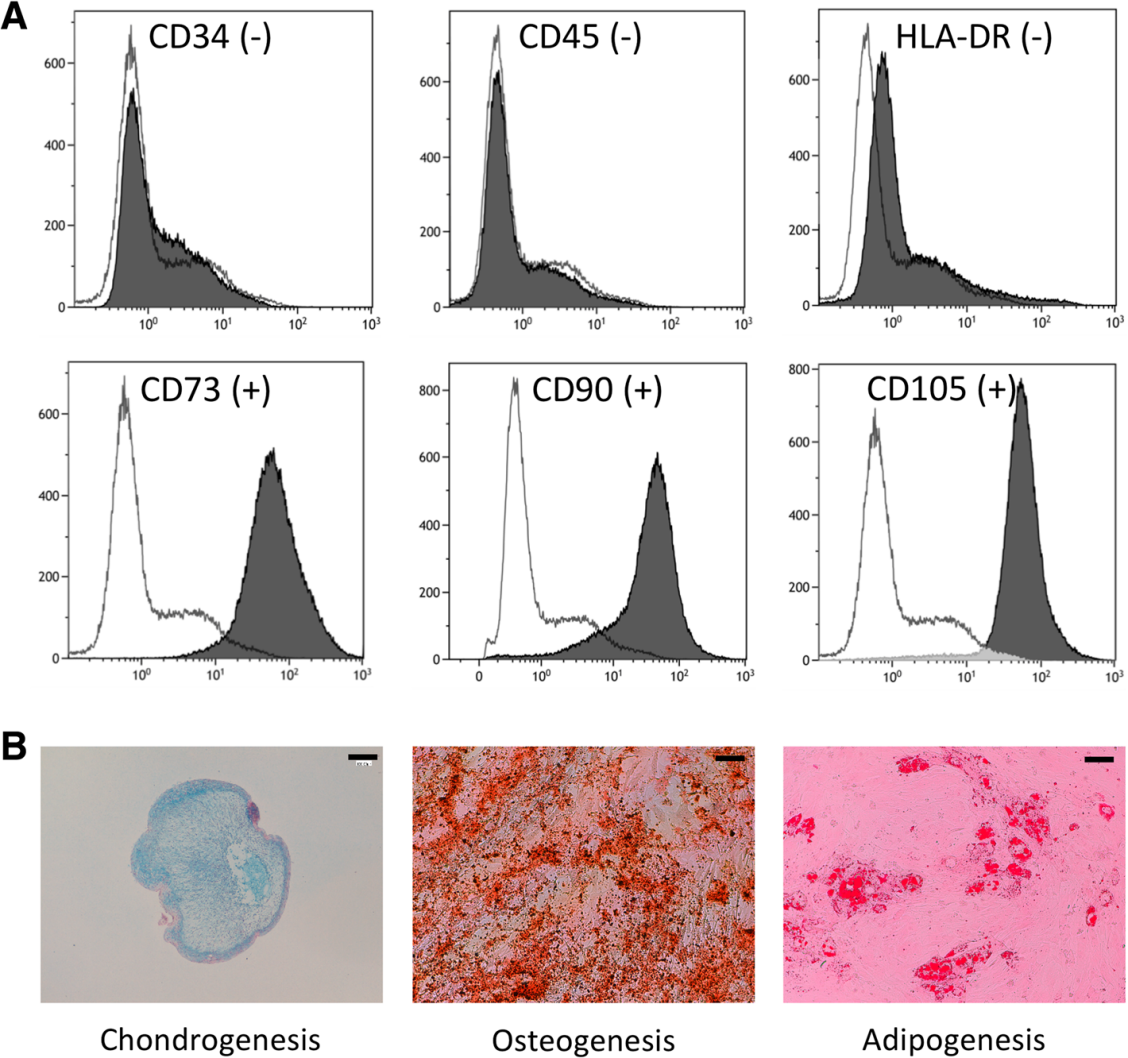
Immunophenotype and trilineage differentiation of fibroblastic cells derived from advanced OA synovial fluid. **a** Surface marker expression of human MSCs derived from advanced OA synovial fluid at passage 2. Each histogram is a representative result of three SF-MSC samples. The white histogram represents negative control response, and black histogram is for the samples. Results showed negativity for CD34, CD45, and HLA-DR and positivity for MSCs markers CD90, CD105, and CD73. **b** Chondrogenic, osteogenic, and adipogenic potentials of SF-MSCs. The chondrogenic differentiation of SF-MSCs was assessed by alcian blue staining on pellet cultured during 28 days in chondrogenic medium (TGF-β1). Cells were cultured in osteogenic medium during 14 days, and calcium mineralization was evaluated by Alizarin Red S staining. Lipid droplets were observed in SF-MSCs cultured in adipogenic induction medium for 28 days with Red Oil staining. A representative example for two samples is shown. Scale bars 200 μm

The characterization of surface epitopes on isolated SF cells was performed at the end of the second passage (P2) demonstrating, as expected [24], that the cells were negative for hematopoietic markers like CD34, CD45, and HLA-DR. Besides, SF-MSCs were strongly positive for CD73 and CD105 and also positive for CD90 (Fig. 1a).

### Multipotency of human SF-MSCs

A pellet culture system was used to evaluate the chondrogenic potential of the SF-MSCs. Histologically, SF-MSCs pellets treated with TGF-β1 exhibited a cartilage matrix synthesis highly stained with alcian blue (Fig. 1b). Additionally, SF-MSC monolayers were cultured in an osteogenic medium in order to evaluate their osteogenic differentiation. After 14 days of culture, we found calcium deposits with alizarin red S staining (Fig. 1b). SF-MSCs were also exposed to an adipogenic induction medium for 21 days and stained with Oil Red O. As expected, lipid vesicles were observed in these SF-MSCs (Fig. 1b).

### Gene profiles in SF-MSCs seeded collagen sponges on D28 under various conditions

#### Chondrogenic genes expression (Fig. 2)

Aggrecan is a major component of the hyaline cartilage. Its gene expression (*ACAN*) is very low under ITS and BMP-2 exposure irrespective of the oxygen tension. In normoxia, only a slight significant *ACAN* overexpression (4.3-fold) was observed under TGF-ß1 and TGF-ß1 plus BMP2. On the other hand, under hypoxia, TGF-ß1 alone (38.9 fold) or in combination with BMP-2 (53.7-fold) significantly strongly increased *ACAN* gene expression.

**Fig. 2:**
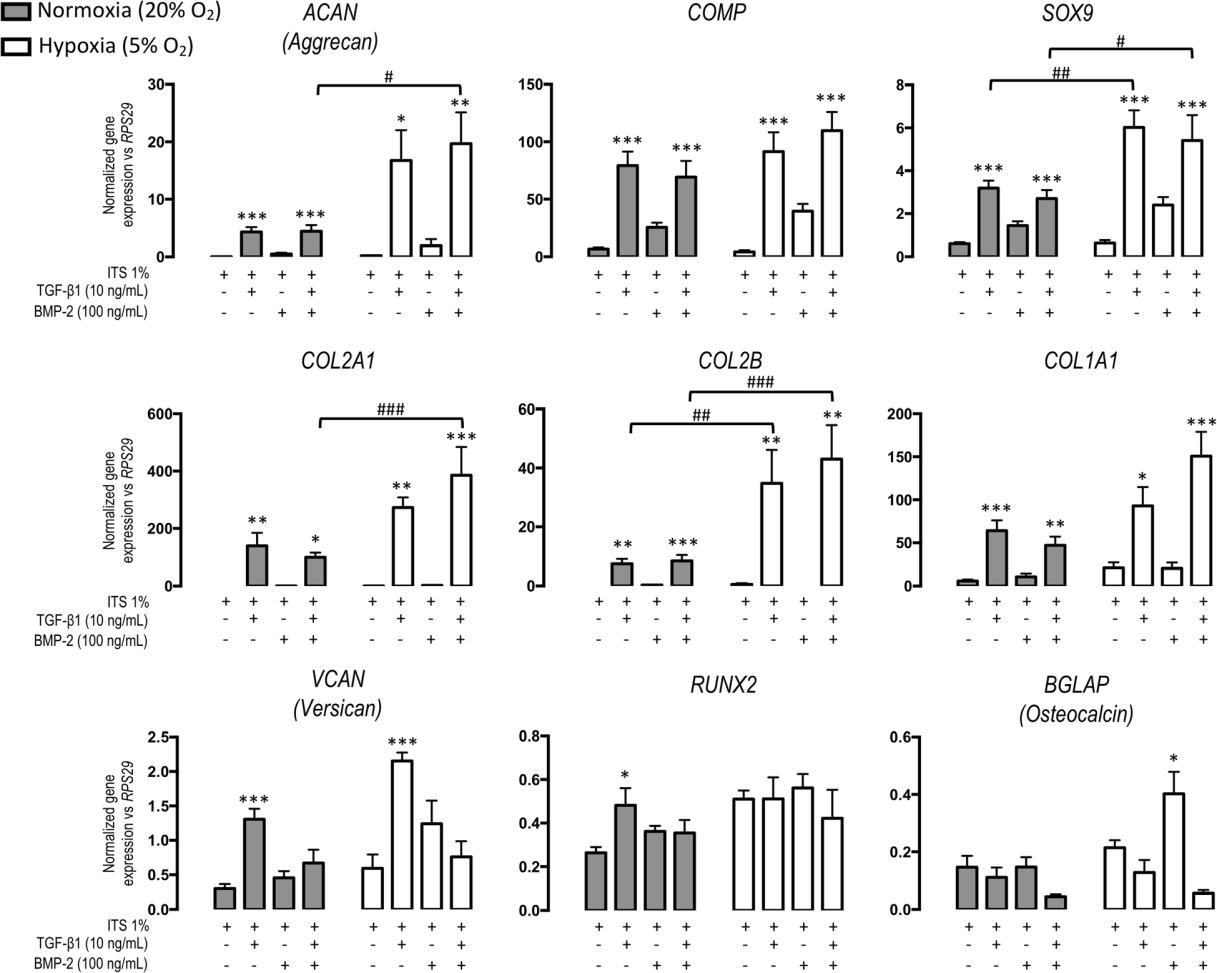
Effect of culture conditions on the mRNA relative expression in cartilage engineered substitutes. Synovial fluid derived mesenchymal stem cells (sf-MSCs) were cultured in collagen sponges in normoxia (21% O2—gray histogram) or in hypoxia (5% O2—white histogram) during 28 days. Sponges were treated from day 3 with ITS 1% as control condition or with TGF-β1 (10 ng/mL) alone or in combination with BMP-2 (100 ng/mL) and BMP-2 (100 ng/mL) alone. We investigated relative mRNA expression by using real-time polymerase chain reaction of chondrogenic (COMP, ACAN, SOX9, COL2A1, COL2B), fibrotic (VCAN, COL1A1) and osteogenic markers (BGLAP and RUNX2). All results were normalized to RPS29 mRNA expression. Each histogram represents three independent experiments performed in triplicate. Data are presented as mean ± standard error of the mean. Statistical analysis was performed initially with a one-way ANOVA with a Dunnett’s post hoc test versus their internal control ITS for each condition (normoxia separated from hypoxia) followed by a two-way ANOVA with a Bonferroni’s post hoc test on all the values the interaction of hypoxia. Asterisks represent significant difference versus the control condition (ITS 1%) *p < 0.05; **p < 0.01; ***p < 0.001 in each condition. Hash sign represents significant difference of growth factor condition in normoxia versus hypoxia taken into account both conditions #*p* < 0.05; ##*p* < 0.01; ###*p* < 0.001 (significant interaction of hypoxia)

*COMP* (cartilage oligomeric matrix protein) is a marker of the hyaline cartilage involved in the assembly of the extracellular matrix. The *COMP* expression, in the ITS condition, was very low for both oxygen tensions. TGF-ß1 alone or in association with BMP-2 strongly increased the COMP expression (79.27 and 69.3) similarly in both oxygen tensions. Again, BMP-2 alone did not significantly boost its expression.

*SOX9* is a transcription factor involved in the chondrocyte differentiation and the cartilage formation. TGF-ß1 induced a slight significant increase (3.2-fold) of *SOX9* expression in normoxia. Hypoxia significantly boosted this overexpression (6.0-fold). Moreover, TGF-ß1 + BMP-2 under normoxia and hypoxia, significantly increased *SOX9* expression (2.7- vs 5.4-fold).

Type II collagen, and particularly its IIB isoform, is specific of hyaline cartilage. Under normoxia, with TGF-ß1 and TGF-ß1 + BMP-2, a significant increase was observed for *COL2A1* versus ITS. Hypoxia exerted a significant influence by significantly enhancing *COL2A1* expression for TGF-ß1 alone (272.8-fold) or combined with BMP-2 (385.4-fold) conditions. A similar profile was observed for IIB isoform; hypoxia significantly potentiated the induction of this gene expression for TGF-β1 (34.8-fold) and TGF-ß1 + BMP-2 (43.0 fold) conditions. BMP-2 alone was inefficient.

#### Fibrogenic genes expression

Type I collagen is present in fibrous tissues like fibrocartilage. Under normoxia, we observed a slight significant increase of *COL1A1* expression with TGF-β1 (64.0-fold) and TGF-β1 + BMP-2 47.1). Besides, under hypoxia, a significant increase of *COL1A1* expression was induced by TGF-β1 (92.9-fold) and TGF-β1 + BMP-2 (150.1-fold). BMP-2 alone was inefficient.

Versican is a proteoglycan, which confers a fibrous character to its surrounding structure. Only TGF-β1, for both oxygen tensions, led to a significant increase of *VCAN* expression (1.3- versus 2.5-fold). No significant influence of the oxygen tension was observed on this week expression.

#### Osteogenic gene expression

Runx2 is a transcription factor involved in osteogenic differentiation. TGF-ß1 alone significantly enhanced its very weak expression. Additionally, hypoxia did not influence *RUNX2* gene expression. Interestingly, osteocalcin gene (*BGLAP*), a specific protein for bone tissue revealing bone terminal differentiation, was only enhanced under hypoxia for the BMP-2 condition (but with a very low fold).

### Histology, immunohistochemistry, and GAG content of cartilaginous tissue-engineered substitutes on D28

#### Histology

**Fig. 3:**
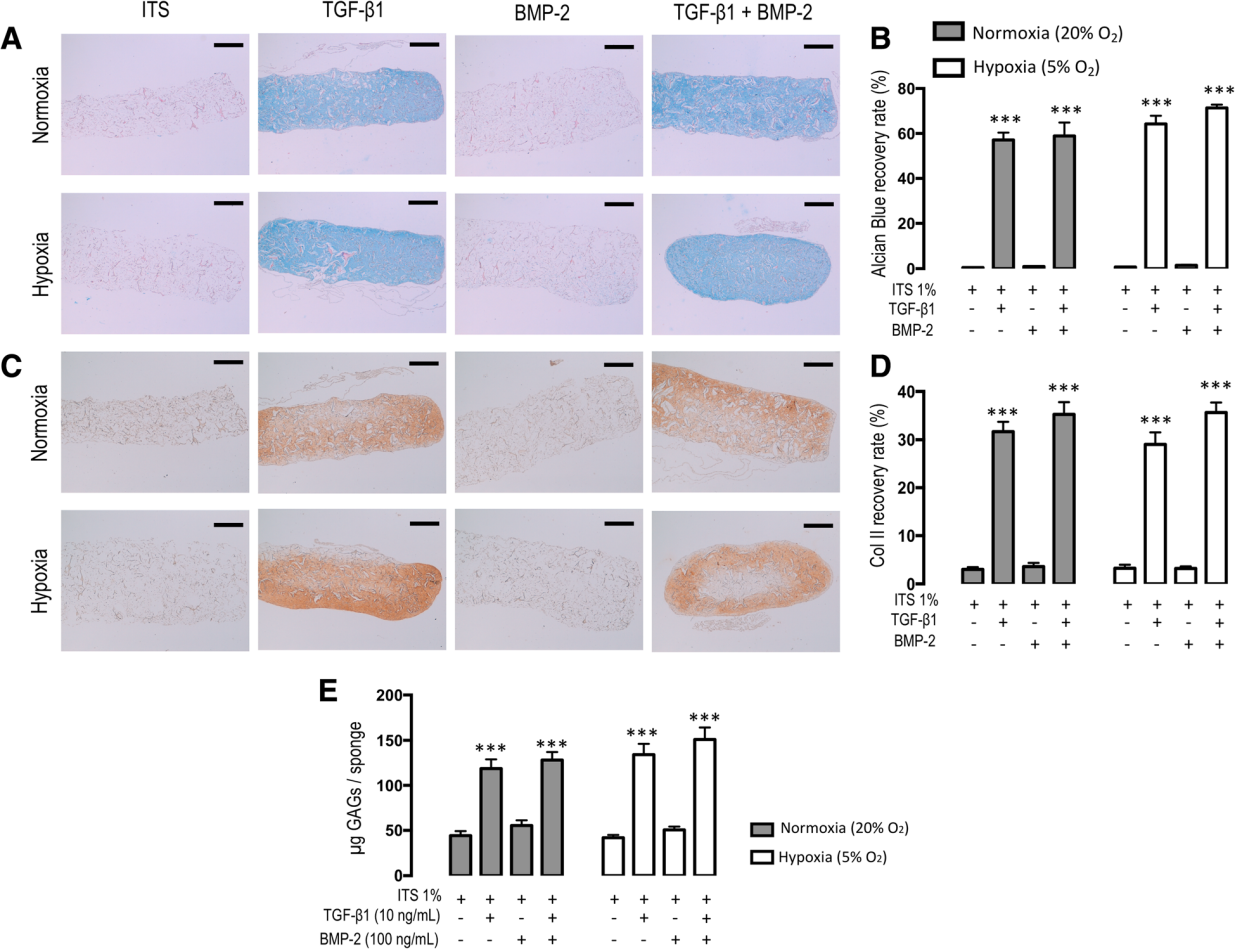
Histological, immunohistochemical analyses and GAG contents of cartilage engineered substitutes produced using human SF-MSCs and collagen sponge at D28 under various oxygen conditions and various growth factors. All observations were carried out on three different sponges for each culture condition and for each patient. The differences between three sponges of each group are very low; we choose to submit only one photograph. The sections were stained with alcian blue to reveal proteoglycan content (**a**) and immuno-histochemical analyses against type II collagen (Col2) was performed (**c**). The scale bars represent 400 μm. **b** and **d** represents densitometry measurement of alcian Blue and type II collagen respectively. Concentration of GAGs was measured inside sponges with a colorimetric assay using dimethylmethylene blue (**e**). Statistical analysis was performed initially with a one-way ANOVA with a Dunnett’s post hoc test versus their internal control ITS for each condition (normoxia separated from hypoxia) followed by a two-way ANOVA with a Bonferroni’s post hoc test on all the values the interaction of hyopoxia. Asterisks represent significative difference versus the control condition (ITS 1%) *p < 0.05; **p < 0.01; ***p < 0.001 (**c, d**, and **e**). There is no significant interaction of hypoxia

Hematoxylin-eosin-safran staining revealed no alteration of the cells. The cellular distribution within the sponges was homogeneous, and no mortality was observed for the different conditions studied (data not shown). On D28, alcian blue (Fig. 3a, b) did not show any matrix synthesis for the ITS control (0.5%) and BMP-2 alone (0.97%, NS vs ITS) in normoxic conditions. TGF-β1 alone or in combination with BMP-2 significantly increased the proteoglycan content in the sponges with a similar fold (Fig. 3b, respectively 57.1% and 58.9%, *p* < 0.001 vs ITS). No significant interaction of oxygen tension was noted by densitometry measurement, as a similar profile was noted in hypoxia.

#### Immunohistochemistry (IHC)

A similar profile was observed on D28 for IHC with type II collagen labeling (Fig. 3c, b). No induction of matrix synthesis by ITS and BMP-2 alone was noted (3.4% vs 3.6%, respectively, NS), while TGF-β1 alone or in combination with BMP-2 induced significantly a strong synthesis similar to the hyaline cartilage ECM (31.7% and 35.3% respectively, *p* < 0.001 vs ITS). Again, the densitometry measurements did not show any significant interaction of hypoxia versus normoxia.

#### GAG content

As shown in Fig. 3e, under normoxia, the GAG content was low for both ITS and BMP-2 conditions (44.2 μg and 55.5 μg, respectively, NS). Similarly, TGF-β1 alone or in combination with BMP-2 significantly enhanced GAG content in collagen sponges (respectively 118.5 μg and 150.9 μg, *p* < 0.001 vs ITS). Evenly, there was no significant interaction of hypoxia versus normoxia on the GAG content.

### Scaffold-free intra-articular injection of SF-MSCs in ACLT model

**Fig. 4:**
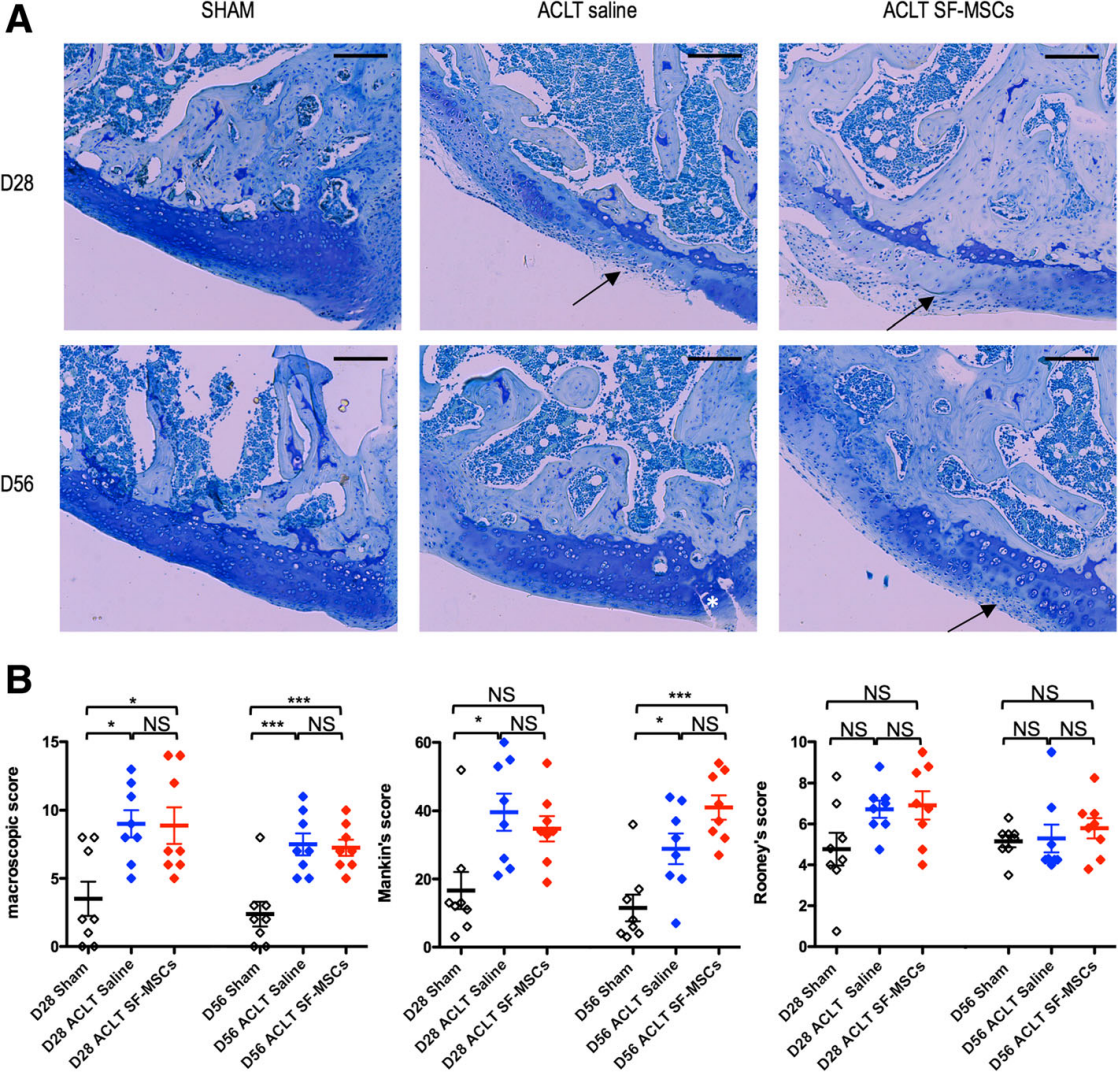
Histological and macroscopic scoring of experimental OA rat model induced by anterior cruciate ligament transection (ACLT) at days 28 and 56 after surgery. **a** Histological evaluation of medial femur by toluidine blue staining. Arrow represents surface irregularities or decrease of matrix contents, asterisk indicate fissure. Scale bars represent 200 μm. **b** represents macroscopic, Mankin’s, and Rooney’s scores of whole studied right knees. Individual values and mean ± SEM from eight rats per batch are represented (D28 and D56 SHAM in black; D28 and D56 ACLT + saline in blue; D28 and D56 SF-MSCs in red). Statistical analysis was performed by a one-way ANOVA with a Bonferroni’s post hoc test for multiple comparisons. Asterisks represent significant difference *p < 0.05; ***p < 0.001

For both times studied (D28 and D56), sham-operated right knees (*n* = 8 + 8) and naive left knees (*n* = 8 + 8) displayed a similar scoring in terms of macroscopy, OA, and synovial scores (data not shown). This means the knee sham surgery and the two i.a. saline injections were not phlogistic and did not interfere with the natural articular growth. Besides, ACLT procedures induced a significant increase in macroscopic and Mankin’s scores on D28 and D56 (Fig. 4). On another note, experimental OA did not generate a significant synovitis, which usually characterizes this model, particularly in the rat. This is probably inherent to the breed used for this study, a T cell-deficient RNU rat, and to its depleted cell populations in thymus-dependent areas of peripheral lymphoid organs being anti-inflammatory in nature. This also probably contributes to the fact that our articular lesions were less severe when compared with our previous practice in historical records of experiments during ACLT-induced knee OA in Wistar rats. Furthermore, i.a. injections of SF-MSCs had no beneficial or detrimental influence on OA lesions and synovial scorings.

## Discussion

We demonstrated herein that end-stage OA synovial fluids obtained from knee joints contained adhering cells undergoing trilineage differentiation in vitro. Most of these cells expressed CD73, CD90, and CD105 and showed very low expression CD34, CD45, and HLA-DR, thus consistent with the phenotype of MSCs [11]. Furthermore, these SF-MSCs, when seeded in collagen sponges, exerted a chondrogenic potential under the influence of TGF-ß1 in terms of gene expression and matrix production (GAGs and type II collagen). BMP-2 alone was ineffective. Surprisingly, hypoxia exerted little to no influence on TGF-ß1 triggered chondrogenicity. Finally, SF-MSCs, when injected intra-articularly during ACLT-induced OA in nude rat knee, are ineffective on structural OA-induced lesions.

Since the sixties, it has long be known that SF from arthritic patients of various conditions, including OA, contains cells capable of in vitro proliferation, but their phenotypic characterization and multipotentiality were demonstrated in 2004 [25]. Their origin has been debated: blood irrigation, shedding from synovium [26], especially type B synovial lining, disrupted superficial cartilage layer [27], subchondral bone, infra-patellar pad [28]. For a decade, the presence of SF-MSCs in normal SF, increasing in early human OA, has been demonstrated [8]. Physiologically, the cartilage homeostasis results from a balance between anabolism and catabolism. With this in mind, SF-MSCs are commonly thought to be originated from synovial tissue [29–31]. Furthermore, SF-MSCs gene expression profile is closely related to cells derived from the synovial membrane [5, 12, 14, 32]. SF MSCs can participate to spontaneous cartilage regeneration at the damaged sites of limited chondral defects, or in ligament repair, in both clinical and experimental conditions, under the influence of cytokines and intra-articular growth factors [33] linked to the underlying pathological condition.

SF microenvironments can thus influence the expression of SF-MSC surface markers [34]. Our patients are all suffering from advanced OA requiring total knee joint replacement, and can be considered as a homogeneous group. In contrast, previous studies have demonstrated that the stage of the disease influences the chondrogenic capacity and the number of SF-MSCs, either experimentally [35, 36] or clinically [6, 12]. In our experimental conditions, archetypal cell-surface makers were positive: CD73, CD90, and CD105, combined with a negative immuno-reactivity for CD34, CD45, and HLA-DR as previously published [11]. These markers can be used to identify a homogeneous population of MSCs, although there are some divergences in the literature. CD105 has been promoted to select SF-MSCs with immuno-magnetic separation [37], as it binds TGF-ß1 and TGF-ß3, but do not predict the chondrogenic potential of MSCs [38]. CD105 and CD90 are present on MSC surface and are lost during differentiation [39]. In addition, CD90+ MSCs predict a good chondrogenic potential [40, 41]. Besides, CD73 expression was observed [6, 9, 42] during synovium and SF MSCs expansions. Additionally, we confirmed that OA SF-MSCs had capabilities to differentiate into adipogenic, osteogenic, and chondrogenic lineages in agreement with the literature [5, 9, 10].

To date, the chondrogenicity of SF-MSCs was mostly studied within a pellet system for the proof concept of multipotency, but little has been reported to study the chondrogenic differentiation of SF-MSCs seeded 3D tissue-engineering constructs, e.g., collagen sponges. In fact, pellet system is a 3D structure based on a cell-cell interaction, while collagen sponge is a porous biomaterial allowing extracellular matrix synthesis. TGF and BMP are prone to enhance chondrogenesis of SF-MSCs [20, 43]. As well, the influence of hypoxia has been rarely studied [20]. Our results confirm the boosting effect of TGF-ß1 on some chondrogenic gene expression (*ACAN*, *COL2A1*, *COMP*, *SOX9*) and extracellular matrix production (GAG, type II collagen) in SF-MSCs seeded collagen sponges on D28. Surprisingly, *ACAN* and *COL2A1* genes were weakly overexpressed during TGF-ß1-driven 3D differentiation under normoxia. In a previous work conducted under normoxia with TGF-ß1-driven differentiation of bone marrow MSCs-seeded collagen sponges, we noticed a highly significant genic over-expression of global type II collagen (495-fold versus ITS condition) and aggrecan (105.6-fold versus ITS condition) [44]. We also observed that BMP-2 alone did not induce SF-MSC chondrogenic differentiation. In combination with TGF-ß1, it did not enhance chondrogenic gene expression. In a similar study carried out on human cord blood MSCs-seeded sponges under normoxia for 14 days, the cocktail TGF-ß1 + BMP-2 significantly upregulated *COL2A1* and *ACAN* [45]*.* As previously published in alginate beads [19] and collagen sponges [46], or in a scaffold-free manner [20], hypoxia promoted chondrogenic gene expression. In contrast, in our experimental conditions, low oxygen tension was without influence at the protein level (histology, immunohistochemistry, and GAG content). Interestingly, osteogenic genes were not significantly highly overexpressed, and there was no calcification in the tissue-engineered constructs.

One possible explanation of this peculiar chondrogenic commitment may be the physio-pathological microenvironment of the SF-MSCs used herein; all harvested in end-stage OA knees undergoing total knee joint replacement. In degenerative conditions, there is mainly a catabolic environment with a significant increase of TNFα and a significant decrease of TGF-ß1 [28]. During OA, synovitis causes hypoxia [47], hypercapnia, and acidity in synovial fluid [48]. In addition, an increase in intra-articular SF volume causes a shift of local metabolism towards anaerobic glycolysis, thus contributing to the decrease of SF glucose levels [49]. Besides, synovial cavity damages correlates with fluctuating oxygen pressure in the joint, overproduction of free radical and lack of oxygen-processing enzymes and free radical scavenging molecules [50, 51]. Moreover, SF-MSCs are exposed to abnormal physical load in biomechanically OA-compromised joints [52]. This is very different in other studies conducted with bone marrow, Wharton’s jelly, and adipose MSCs, all being resident in physiological conditions. In fact, intra-articular exposure to these pro-inflammatory agents [53] may either realize a SF-MSCs preconditioning (or priming) or alter their paracrine properties, thus accounting for their particular commitment [34].

To date, there is no published clinical study regarding SF-MSC-based cartilage regeneration [54] and very few in vivo experimental studies are available [54], especially in rodents. To the best of our knowledge, one xenogenic study has been conducted in the rat knee (2-mm-diameter articular defect in trochlear groove) by using equine SF-MSC-seeded agarose constructs. This study was successful in terms of in vivo evaluation and type II collagen expression [55]. Besides, human SF-MSCs (mild OA, arthroscopic flushing) encapsulated in a one-step rapid cross-linked hydrogel significantly ameliorates cartilage defects in rat knee after 8 weeks [56]. Another study was conducted in porcine knee chondral defects with autologous porcine SF-MSCs mixed with platelet-rich plasma and thermosensitive hydrogel, demonstrating a great ability for chondrocyte regeneration and maturation at 4 and 8 weeks after implantation [57]. Similar results were reported in rabbits with autologous SF-MSCs [58]. No experimental protocol has yet been conducted with SF-MSCs in experimental diffuse (ACLT) induced OA. One recent work, conducted with synovial membrane-MSCs during ACLT in the Lewis rat showed that a single injection was ineffective but weekly injections significantly improve OARSI score at 8 and 12 weeks [59]. A previous work in mono-iodoacetate-induced OA showed that the priming with inflammatory factors did not influence the migration or adhesion capacity of human MSCs to OA synovium after intra-articular injection in the rat knee [34]. During this model of diffuse OA, intra-articular injections of allogenic bone marrow MSCs were effective on pain but not on OA lesions [60]. Cartilage repair thus seems a better indication than diffuse OA for synovial tissue derived MSCs. Accordingly, allogenic rat synovium-derived MSCs have been successfully intra-articularly injected for an osteochondral defect model in the femoral groove in Sprague-Dawley rats [61]. Similar beneficial results were reported in C57BL6 mice with focal knee lesions with a single injection of allogenic synovium MSCs [62].

We used nude rats to avoid an adverse response after repeated intra-articular injection of xenogenic mesenchymal stem cells, which was previously observed with allogenic MSCs [63]. This breed has already been used to assess the success of a third-generation human chondrocyte implantation in a mixed model combining ACLT and knee focal defect in the patella groove [64]. Two weeks after ACLT, OA lesions were depicted macroscopically. Interestingly, human MSCs injected intra-articularly were chondroprotective in a compartmental OA model in nude rat knee induced by medial meniscectomy [65]. Similar results were reported with human-induced pluripotent stem cell injected in a calibrated focal lesion performed in immuno-compromised rat knees [66]. Nude rats were also used to evaluate the regenerative properties of muscle-derived MSCs in the MIA model [67, 68]. In all these studies, none paid attention to synovitis. We were surprised by the low-grade knee synovitis in our experimental conditions in the control group (saline), contrasting with our previous experience in Wistar rats, assessed by histology and MRI [23, 69, 70]. Nevertheless, OA scoring was significantly more severe in ACLT-OA group versus sham group. A blessing in disguise, intra-articular injections of human SF-MSCs did induce neither chondroprotective effect, nor phologistic/metaplasic reactions in the synovium. Similarly, no synovitis was depicted in knee nude rats OA induced by a meniscal tear on D28 [71]. Nevertheless, as previously described in Wistar rats knees by Leijs [34], describing no significant difference in human bone MSCs attachment to synovium between OA (MIA) and healthy rat knees, this may suggest that a previous synovitis is not an obligatory prerequisite to activate exogenous MSC’s trophic properties after intra-articular injection or implantation. Additionally, the number of MSCs injected have been determined according to the literature, although there is no real consensus on the ideal dose and timing of injections [72]. After intra-articular injection and homing, MSCs probably exert their symptomatic beneficial analgesic properties [60, 73], at least, by their paracrine anti-inflammatory and anti-apoptotic activities, rather than a structural articular protection.

## Conclusion

The use of SF-MSCs is advantageous due to their easy harvesting during arthrocentesis or arthroscopy. The number of SF-MSCs is low in physiological conditions but notably increases in osteoarthritic conditions. In this study, we have characterized the stemness and demonstrated the multilineage potency of human advanced OA SF-MSCs. Furthermore, the chondrogenic induction (TGFß1 alone or in combination with BMP2) of these SF-MSCs in collagen sponges demonstrates a good capacity of chondrogenic gene induction and extracellular matrix synthesis. Surprisingly, probably due to previous priming by inflammatory SF microenvironment, hypoxia did not enhance matrix synthesis, although it boosted chondrogenic gene expression (*ACAN*, *SOX9*, *COL2A1*). This is a promising result for cell-based constructs dedicated for cartilage defects. Besides, scaffold-free intra-articular injections of xenogenic SF-MSCs did not produce any beneficial or detrimental influence on articular lesions in ACLT-induced OA in the nude rat knee.

## Abbreviations

3D: Three-dimensional
AB: Alcian blue
ACAN: Aggrecan
ACI: Autologous chondrocyte implantation
ACLT: Anterior cruciate ligament transection
ACP: Articular cartilage progenitor
bFGF: Basic fibroblast growth factor
BGLAP: Osteocalcin
BMP-2: Bone morphogenetic protein-2
BSA: Bovine serum albumin
CD: Cluster differentiation
cDNA: Complementary deoxyribonucleic acid
COL1A1: Type I collagen
COL2A1: Type II collagen
COL2B: Type II B collagen
COMP: Cartilage oligomeric matrix protein
D28: Day 28
DAB: Diaminobenzidine
DMB: Dimethylblue
DMEM-HG: Dulbecco’s modified Eagle’s medium-high glucose
DMEM-LG: Dulbecco’s modified Eagle’s medium-low glucose
ECM: Extracellular matrix
FBS: Fetal bovine serum
GAGs: Glycosaminoglycans
HES: Hematoxylin, eosin, and saffron stain
HLA-DR: Human leukocyte antigen - antigen D related
HSB: Hue, saturation and brightness
i.a.: Intra-articular
IHC: Immunohistochemistry
ITS: Insulin, transferrin, selenium
mRNA: Messenger ribonucleic acid
MSCs: Mesenchymal stem cells
NaCl: Sodium chloride
OA: Osteoarthritis
P2: Passage 2
P3: Passage 3
P4: Passage 4
PBS: Phosphate-buffered saline
PFA: Paraformaldehyde
RGB: Red, green and blue
RNA: Ribonucleic acid
ROI: Region of interest
RPS29: Ribosomal protein S29
RT-PCR: Reverse transcription polymerase chain reaction
RUNX2: Runt related transcription factor 2
SF: Synovial fluid
SF-MSCs: Synovial fluid derived mesenchymal stem cells
SOX9: Sex determining region Y-box 9
TGF-β1: Transforming growth factor-β1
TNFα: Tumor necrosis factor alpha
VCAN: Versican
